# Removal of a torn biliary stent fragment using a novel tapered-tip sheath system

**DOI:** 10.1055/a-2304-8328

**Published:** 2024-04-29

**Authors:** Kenji Sawada, Tomoaki Matsumori, Yoshihiro Nishikawa, Takahisa Maruno, Masahiro Shiokawa, Norimitsu Uza, Hiroshi Seno

**Affiliations:** 1 Department of Gastroenterology and Hepatology, Kyoto University Graduate School of Medicine, Kyoto, Japan

A 64-year-old woman with a severe benign biliary stricture, resulting from a biliary fistula after a right anterior segmentectomy for liver metastasis from colonic cancer, underwent endoscopic retrograde cholangiopancreatography for a biliary inside stent exchange ( Fig. 1 ). Initially, we attempted stent removal using grasping forceps; however, the thread attached to the stent broke, making grasping difficult ( Fig. 2 ). Furthermore, the severe benign stricture prevented dilation with a balloon catheter. Despite successful removal of most of the inside stent using a snare catheter, the distal tip of the stent remained on the peripheral side of the stricture ( Fig. 3 ). Subsequently, a novel device (EndoSheather; Piolax, Kanagawa, Japan) comprising a tapered-tip inner catheter and an outer sheath with a coaxial two-layer structure was deployed ( Fig. 4 ) 1 2 . The device penetrated the stricture smoothly and the radiopaque marker on the outer tip of the sheath indicated that the device was situated below the fragment ( Fig. 5 **a** ). The fragment was then removed successfully using biopsy forceps that were inserted through the outer sheath of the device ( Fig. 5 **b** – **d** , Video 1 ).

**Fig. 1:**
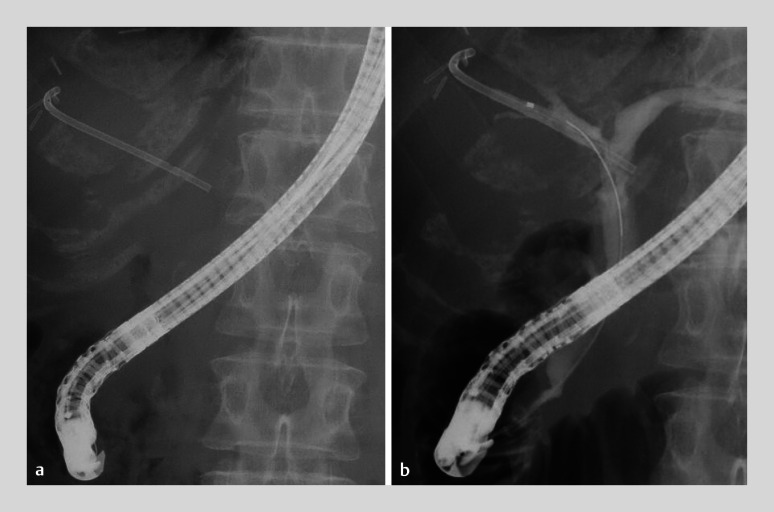
Fluoroscopic images showing placement of a plastic biliary inside stent for a benign biliary stricture resulting from a biliary fistula after right anterior segmentectomy.

**Fig. 2:**
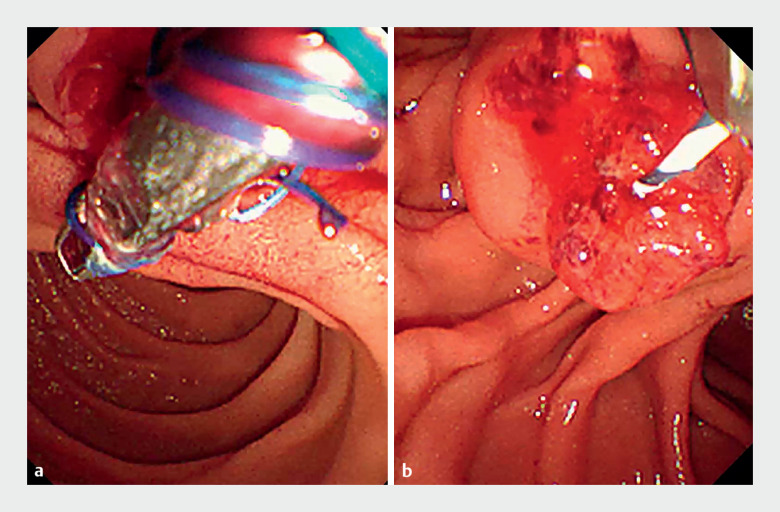
Endoscopic images showing: **a** attempted removal of the inside stent using grasping forceps; **b** the broken thread of the stent, which made it challenging to grasp.

**Fig. 3:**
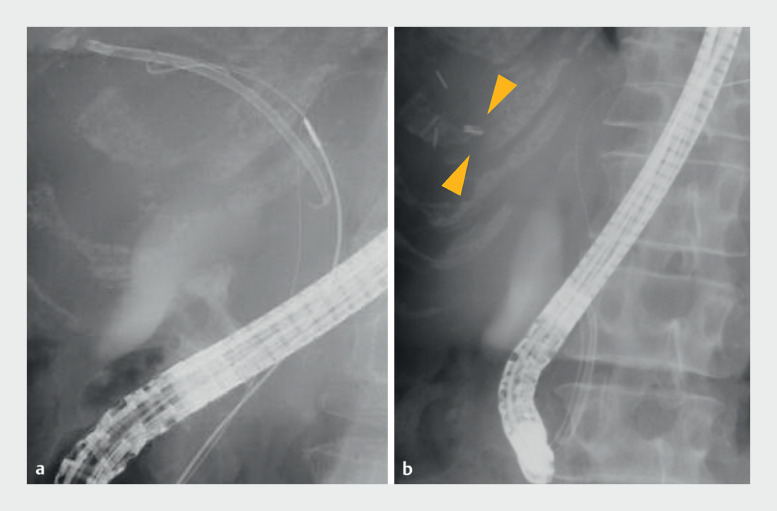
Fluoroscopic images showing: **a** insertion of a snare catheter for direct stent retrieval; **b** a fragment of the stent that was left behind on the peripheral side of the stricture (yellow arrows).

**Fig. 4:**
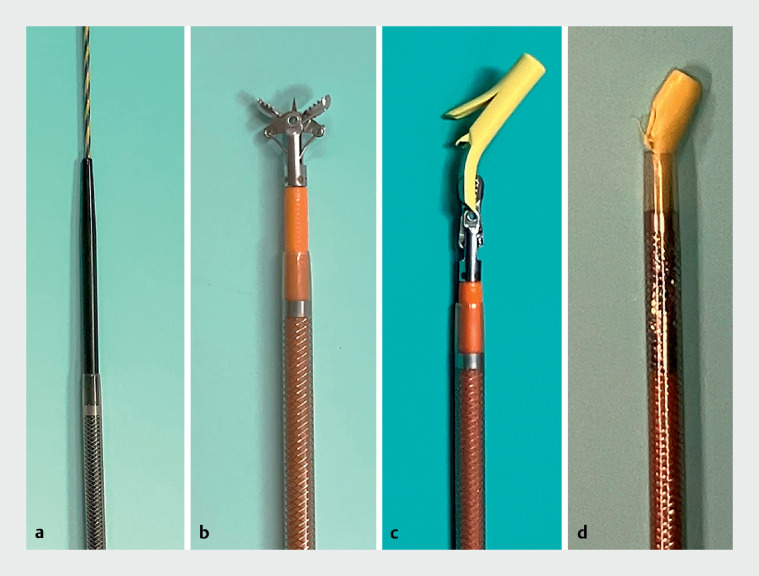
Photographs of the novel device showing: **a** the guidewire passing through the inner catheter; **b** biopsy forceps inserted through the outer sheath after removal of the inner catheter and guidewire; **c** the coaxialized stent fragment, biopsy forceps, and outer sheath of the device; **d** the stent fragment dragged back into the outer sheath of the novel device.

**Fig. 5:**
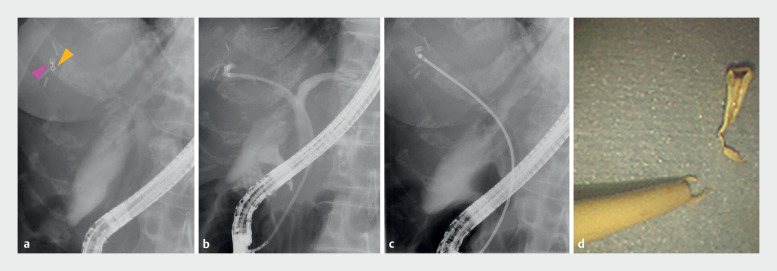
Fluoroscopic images ( **a** – **c** ) showing: **a** the radiopaque marker at the tip of the outer sheath of the device (yellow arrow) positioned just below the stent fragment (pink arrow); **b** the narrow lumen on the peripheral side of the biliary stricture; **c** biopsy forceps inserted through the outer sheath to grasp the stent fragment. **d** Photograph of the removed stent fragment.

A novel device is used to allow capture of a broken biliary inside stent fragment beyond a biliary duct stricture.Video 1

In patients with hilar biliary stricture, inside stents sometimes migrate and break during the removal process, leaving fragments of the stent in the bile duct, from where their removal is technically challenging 3 . Recent reports have described the removal of foreign bodies from the bile duct using this novel device 4 5 . Notably in this case, the device enabled reliable removal of a floating foreign body in a narrow peripheral bile duct upstream of a severe biliary stricture. This may be a result of the device’s stricture-dilating function and its concomitant ability to pull the foreign body out coaxially, enabling the removal of floating foreign bodies that are difficult to grasp upstream of a biliary stricture, even in situations where the foreign body cannot be retracted into the outer sheath ( Fig. 4 ) 2 .

This device could be useful for the removal of floating foreign bodies on the peripheral side of biliary strictures.

Endoscopy_UCTN_Code_CPL_1AK_2AD

## Notes

Endoscopy E-Videos https://eref.thieme.de/e-videos
*E-Videos* is an open access online section of the journal *Endoscopy* , reporting on interesting cases and new techniques in gastroenterological endoscopy. All papers include a high-quality video and are published with a Creative Commons CC-BY license. Endoscopy E-Videos qualify for HINARI discounts and waivers and eligibility is automatically checked during the submission process. We grant 100% waivers to articles whose corresponding authors are based in Group A countries and 50% waivers to those who are based in Group B countries as classified by Research4Life (see: https://www.research4life.org/access/eligibility/ ). This section has its own submission website at https://mc.manuscriptcentral.com/e-videos .

## References

[1] Matsumori T Uza N Shiokawa M Mapping biopsy for bile duct cancer using a novel device delivery system Endoscopy 2022 54 E217 E219 10.1055/a-1479-1969 34058756

[2] Matsumori T Uza N Shiokawa M Self-expandable metallic stent placement for malignant biliary stricture using a novel device delivery system VideoGIE 2021 6 468 471 10.1016/j.vgie.2021.07.003 34667913 PMC8504182

[3] Panagiotis K Jannis K George P Migration of plastic biliary stents and endoscopic retrieval: an experience of three referral centers Surg Laparosc Endosc Percutan Tech 2009 19 217 221 19542849 10.1097/SLE.0b013e3181a031f5

[4] Yamada M Okamoto T Sasahira N Successful removal of a migrated plastic stent using a new endoscopic sheath Endoscopy 2023 55 E1250 E1251 10.1055/a-2215-1232 38092059 PMC10718943

[5] Mori Y Kurita A Successful retrieval of a fractured biliary guidewire using a newly developed endoscopic tapered sheath Endoscopy 2023 55 E706 E707 10.1055/a-2073-5147 37164336 PMC10172000

